# Accelerated Stochastic Conjugate Gradient for a class of convex optimization

**DOI:** 10.1371/journal.pone.0338720

**Published:** 2025-12-29

**Authors:** Lulu He, Yanan Du

**Affiliations:** 1 School of Mathematics-Physics and Finance, Anhui Polytechnic University, Anhui, China; 2 School of Public Administration, Central China Normal University, Wuhan, Hubei, China; Northwestern Polytechnical University, CHINA

## Abstract

The conjugate gradient method is widely recognized as a foundational technique for large-scale unconstrained optimization. In this work, we introduce an Accelerated Stochastic Conjugate Gradient (ASCG) algorithm, specifically designed for a class of convex empirical risk minimization problems. The proposed ASCG method integrates a variance-reduced gradient estimator-inspired by modern stochastic variance reduction techniques-to control noise and improve stability in the optimization process. Moreover, the ASCG algorithm incorporates a novel acceleration mechanism via a deflation factor on the step size, which is shown to achieve faster practical convergence compared to the baseline stochastic FR method. We provide a rigorous theoretical analysis demonstrating that ASCG achieves an expected linear convergence rate under strong convexity assumptions and attains a superior reduction in function values compared to non-accelerated stochastic counterparts. Extensive numerical experiments on four widely-used benchmark datasets confirm that ASCG consistently outperforms state-of-the-art stochastic optimization methods.

## 1 Introduction

This paper primarily addresses the following empirical risk minimization (ERM) problem:

$$x^{*}\in \underset{x\in {\mathbb{R}}^{d}}{\arg\min}f(x)≜\frac{1}{n}\sum_{i=1}^{n}f_{i}(x)$$ (1)

where *n* is the number of training samples with finite yet extremely large value, and *d* represents the dimensionality of the feature space. In this paper, we assume that the set of optimal solutions of problem (1) is nonempty. It is evident that (1) represents a finite-sum problem, which commonly arises in the fields of statistics and machine learning. In such problems, a set of training data $\left\{(a_{1},b_{1}),...,(a_{n},b_{n})\right\}$ will be given for data fitting. The popular loss function that frequently used, such as, *l*_2_ regularized least-squares with $f_{i}(x)=\frac{1}{2}(a_{i}^{T}x$ − $b_{i}{)}^{2}$  +  $\frac{\lambda }{2}||x|{|}_{2}^{2}$ for regression analysis, as well as *l*_2_ regularized logistic regression with $f_{i}(x)=\log(1$  +  $e^{-b_{i}a_{i}^{T}x})$  +  $\frac{\lambda }{2}||x|{|}^{2}$ for binary classification.

Numerous efficient gradient-based methods have been employed to solve problem (1), including gradient descent and its variants [1,2]. Among these, the conjugate gradient method (CGM) stands out as a particularly notable class of gradient-based optimization techniques, renowned for its superior convergence properties in specific problem types. Originally introduced by Hestenes and Stiefel [3] for solving linear systems, CGM was later extended by Fletcher and Reeves [4] to nonlinear optimization problems, leading to the development of nonlinear CGM. Prominent nonlinear variants include the FR-CGM [4], PR-CGM [5], and DY-CGM [6]. CGM offers two key advantages over other methods: it converges faster than gradient descent and avoids the computational burden of Hessian matrix evaluation required in Newton’s method. As a result, CGM has been widely applied in machine learning domains such as compressed sensing [7], image restoration [8], and signal processing [9]. However, since CGM requires the computation of the full gradient at each iteration, it becomes impractical for problems involving extremely large datasets.

To enhance the applicability of CGM to large-scale unconstrained ERM problems, the randomized CGM was developed. Schraudolph and Graepel [10] were the first to incorporate ideas from CGM into a stochastic setting, experimentally demonstrating convergence rates orders of magnitude faster than stochastic gradient descent. Huang et al. [11] combined a stochastic recursive gradient approach with the Barzilai-Borwein technique to address nonconvex optimization, providing a theoretical convergence analysis and empirical validation in machine learning tasks. Randomized CGM has since been extensively applied across areas including digital predistortion [12], pattern classification [13], seismic inversion [14], neural language modeling [15], and deep learning [16]. Nevertheless, the high variance inherent in stochastic gradient estimates considerably impedes the convergence speed of randomized CGM.

Recent years have witnessed significant progress in variance reduction (VR) techniques, which have effectively addressed the high variance inherent in stochastic gradient estimates and substantially enhanced the convergence guarantees of stochastic optimization algorithms. By employing control variates to correct stochastic gradients, VR methods construct gradient estimators with asymptotically vanishing variance, thereby accelerating convergence both in theory and practice. Pioneering VR-based approaches such as SVRG [17] and SAGA [18] laid the foundation for this line of research. While both target convex empirical risk minimization problems, they diverge in mechanism: SVRG adopts a nested-loop structure that periodically refreshes the full gradient, whereas SAGA maintains a historical gradient matrix to enable incremental updates. Building on these ideas, more recent innovations such as SARAH [19], SPIDER [20], and PAGE [21] have further refined the trade-offs between computational cost, storage, and convergence rate, achieving even

faster convergence under various settings. These advances set the stage for the integration of variance reduction into more complex optimization frameworks, including stochastic conjugate gradient methods.

The integration of VR techniques has opened new avenues for enhancing randomized CGM, leading to significant improvements in convergence efficiency and stability. For instance, Jin et al. [22] effectively combined SVRG with CGM to develop the CGVR algorithm, which achieves linear convergence under strong convexity and smoothness assumptions. In a different direction, Kobayashi and Liduka [23] integrated Adam-type adaptive updating with nonlinear CGM, substantially boosting performance in deep neural network training. Further extending this line of work, Kou and Yang [24] proposed the SCGA method, drawing inspiration from SAGA and FR-CGM, and established its linear convergence for smooth and strongly convex objectives. More recently, Ouyang et al. [25] introduced a variance-aware three-term conjugate gradient method equipped with advanced line search techniques tailored for nonconvex optimization. While these VR-enhanced methods consistently outperform conventional CGM on large-scale empirical risk minimization problems, challenges related to online step-size selection persist. In response, Yang [26,27] proposed innovative solutions using local quadratic approximation and hyper-gradient descent, providing more robust and adaptive strategies for step-size control.

In parallel to advancements in stochastic variants, significant research efforts have been devoted to acceleration techniques aimed at further improving the convergence properties of CGM. Early foundational work by Lenard [28] introduced a family of accelerated CGMs drawing analogies to the Broyden family of quasi-Newton methods, establishing a theoretical bridge between these two classes of algorithms. Building on this, Andrei [29–31] developed accelerated CGM variants that achieve substantial reductions in function values through sophisticated step-size selection and direction updating strategies. More recently, Sun et al. [32,33] derived an acceleration parameter using quadratic interpolation models and introduced specific acceptance criteria to enhance robustness and efficiency in practical implementations. Jian et al. [34] reformulated the conjugate parameter via accelerated subspace quadratic optimization, effectively exploiting structural advantages of linear CGM in large-scale settings. Further extending its applicability, Hu et al. [35] incorporated adaptive momentum into nonlinear CGM, demonstrating notable improvements in convergence for sparse recovery problems. Most recently, Karimi and Vavasis [36] proposed a hybrid C+AG algorithm that integrates Nesterov’s accelerated gradient framework with conventional CGM, achieving optimal performance for quadratic functions while maintaining competitive complexity bounds for general smooth convex functions. Collectively, these contributions not only underscore the enduring theoretical value of acceleration mechanisms in CGM but also greatly expand their practical utility in modern computational environments.

Despite these advances, the development of accelerated stochastic conjugate gradient methods remains relatively underexplored, particularly within the context of variance-reduced optimization. To address this gap, we propose a novel Accelerated Stochastic Conjugate Gradient (ASCG) algorithm that synergistically integrates acceleration mechanisms with stochastic variance reduction. Our approach embeds a conjugate gradient update within a stabilized SVRG-like framework, effectively mitigating gradient noise while preserving directional fidelity. Furthermore, we introduce an adaptive step-size scaling strategy that incorporates an acceleration factor to enhance convergence dynamics without compromising stability. We provide a rigorous theoretical analysis establishing that ASCG achieves an expected linear convergence rate for strongly convex empirical risk minimization problems, alongside a provably superior reduction in function values relative to existing non-accelerated stochastic CGMs. To the best of our knowledge, this work constitutes the first systematic effort to incorporate acceleration techniques into stochastic conjugate gradient frameworks, offering both foundational theoretical insights and tangible practical benefits.

The main contributions of this paper as follows:

- We propose the Accelerated Stochastic Conjugate Gradient (ASCG) algorithm-a novel framework that integrates SVRG-based variance reduction to mitigate stochastic gradient noise and a step size acceleration factor to address online step size inefficiencies of randomized CGM.
- We prove that ASCG achieves an expected linear convergence rate for strongly convex ERM problems, with rigorously derived acceleration guarantees that outperform non-accelerated stochastic CGM in function value reduction.
- Our work fills a critical research gap by being the first to combine VR and acceleration techniques for stochastic CGM, a direction previously unaddressed in existing literature.
- Unifies acceleration techniques with variance reduction for CGM, demonstrated through theoretical analysis to be effective for both regression *l*_2_-regularized least squares) and classification (logistic regression) tasks.

Now we provide a detailed description of the structure of this article. We briefly review some definitions as well as related properties in Sect 2. Sect 3 provides ASCG algorithm in detail. The convergence analysis is given in Sect 4. In Sect 5, we carry out practical experiments to illustrate the superiority of ASCG algorithm. Finally, the conclusion is given in Sect 6.

## 2 Notations and preliminaries

In this paper, we denote an *d*-dimensional Euclidean space by ${\mathbb{R}}^{d}$. We use || · || to define the standard *l*_2_ norm, and let ⟨·,·⟩ be the inner product induced by *l*_2_ norm. We use $domf=\{x\in {\mathbb{R}}^{d}|f(x)<\infty \}$ to denote the domain of an extended-real-valued function $f:{\mathbb{R}}^{d}\to (-\infty ,\infty ]$. We let ∇f(x) be the gradient of a continuously differentiable function *f* at the point $x\in {\mathbb{R}}^{d}$. For an integer *n*, we denote by ⌊n⌋ the largest integer that does not exceed it. For a stochastic algorithm 𝒜, we use 𝔼[·] to denote the total expectation in terms of the whole iteration process of 𝒜. For a certain random variable *i*, we denote its expectation by ${𝔼}_{i}[\cdot ]$.

Below we provide several basic definitions associated with their properties.

**Definition 1.** (L−smooth) *[37, Definition 5.1] A continuously differentiable function* $f:{\mathbb{R}}^{d}\to \mathbb{R}$ *is L-smoothness if and only if*


$$||\nabla f(x)-\nabla f(y)||\leq L||x-y||,\;\forall x,y\in {\mathbb{R}}^{d}.$$

*Then we call that f has a Lipschitz continuous gradient.*

Below is an important property in terms of Lipschitz continuous gradient, which will be used frequently in this paper.

**Lemma 1.** *[38, Theorem 2.1.5] If a continuously differentiable function* $f:{\mathbb{R}}^{d}\to \mathbb{R}$ *has a Lipschitz continuous gradient, then the following holds*


$$f(y)\leq f(x)+\langle \nabla f(x),y-x\rangle +\frac{L}{2}||y-x|{|}^{2},\;\forall x,y\in {\mathbb{R}}^{d}.$$

*If further assume that f is convex, then*


$$\frac{1}{2L}||\nabla f(x)-\nabla f(y)|{|}^{2}\leq f(x)-f(y)-\langle \nabla f(y),x-y\rangle ,\;\forall x,y\in {\mathbb{R}}^{d}.$$

**Definition 2.** *[38, Definitioin 2.1.3] A function* $f:{\mathbb{R}}^{d}\to \mathbb{R}$ *is continuously differentiable and* μ*-strongly convex if*


$$f(x)\geq f(y)+\langle \nabla f(y),x-y\rangle +\frac{\mu }{2}||x-y|{|}^{2},\;\forall x,y\in {\mathbb{R}}^{d}.$$

**Lemma 2.** *[38, Theorem 2.1.5] Suppose that f is continuously differentiable and strongly convex with parameter* μ. Let x* *be the unique minimizer of f. Then*


$$||\nabla f(x)|{|}^{2}\geq 2\mu (f(x)-f(x^{*})),\;\forall \;x\in {\mathbb{R}}^{d}$$

*holds.*

## 3 The description of ASCG algorithm

This section provides the main ideas of ASCG algorithm in detail. We begin with introducing the following update rule of CGM:


$$\{d_{k}=-g_{k}+\beta_{k}d_{k-1},\;x_{k+1}=x_{k}+\alpha_{k}d_{k},$$

where *g*_*k*_ defines the gradient of the objective function at the current point *x*_*k*_ and $\beta_{k}$ is the conjugate parameter. The stepsize $\alpha_{k}$ can be determined by line search, such as Armijo condition and standard Wolfe condition. A commonly used more enhanced condition, i.e., the strong Wolfe condition:


$$\{f(x_{k}+\alpha_{k}d_{k})\leq f(x_{k})+\rho \alpha_{k}g_{k}^{T}d_{k},\;|g_{k+1}^{T}d_{k}|\leq -\sigma g_{k}^{T}d_{k},$$

where the parameters 0<ρ<σ<1. The first condition is called sufficient decrease condition, and the second condition is called the curvature condition. To solve problem (1) with a stochastic method, we introduce the random strong Wlofe condition. Assume that we randomly sampling $S_{k}\subset \{1,2,...,n\}$ at the current point *x*_*k*_, then we have the following update rule:

$$\{f_{S_{k}}(x_{k}+\alpha_{k}d_{k})\leq f_{S_{k}}(x_{k})+\rho \alpha_{k}g_{k}^{T}d_{k},\;|g_{k+1}^{T}d_{k}|\leq -\sigma g_{k}^{T}d_{k},$$ (2)

where $f_{S_{k}}(x_{k})=\frac{1}{\left|S_{k}\right|}\sum_{i\in S_{k}}f_{i}(x_{k})$ and *g*_*k*_ are some stochastic gradient at the point *x*_*k*_.

For realizing the acceleration, let we introduce a variable $z_{k}^{s}=x_{k}^{s}+\alpha_{k}d_{k}^{s}$ (see the following Algorithm 1). It follows from the first Wolfe condition in (2) that

$$f_{S_{k}}(z_{k}^{s})=f_{S_{k}}(x_{k}^{s}+\alpha_{k}d_{k}^{s})\leq f_{S_{k}}(x_{k}^{s})+\rho \alpha_{k}(v_{k}^{s}{)}^{T}d_{k}^{s}.$$ (3)

where $v_{k}^{s}$ is the variance reduced stochastic gradient (see the step 14 in Algorithm 1 below). Let we introduce a new variable $x_{k+1}^{s}=x_{k}^{s}+\eta \alpha_{k}d_{k}^{s}$, where η>0 is a parameter which follows to be determined in such a way as to improve the behavior of the algorithm. Now we have

$$f(z_{k}^{s})=f(x_{k}^{s})+\alpha_{k}\nabla f(x_{k}^{s}{)}^{T}d_{k}^{s}+\frac{1}{2}\alpha_{k}^{2}(d_{k}^{s}{)}^{T}\nabla^{2}f(x_{k}^{s})d_{k}^{s}+o(||\alpha_{k}d_{k}^{s}|{|}^{2}),$$ (4)

and

$$f(x_{k+1}^{s})=f(x_{k}^{s})+\eta \alpha_{k}\nabla f(x_{k}^{s}{)}^{T}d_{k}^{s}+\frac{1}{2}\eta^{2}\alpha_{k}^{2}(d_{k}^{s}{)}^{T}\nabla^{2}f(x_{k}^{s})d_{k}^{s}+o(||\eta \alpha_{k}d_{k}^{s}|{|}^{2}).$$ (5)

By combining the Eqs (4) and (5), we obtain

$$f(x_{k+1}^{s})=f(z_{k}^{s})+\Psi_{k}^{s}(\eta ),$$ (6)

where $\Psi_{k}^{s}(\eta )=\frac{1}{2}(\eta^{2}-1)\alpha_{k}^{2}(d_{k}^{s}{)}^{T}\nabla^{2}f(x_{k}^{s})d_{k}^{s}+(\eta -1)\alpha_{k}\nabla f(x_{k}^{s}{)}^{T}d_{k}^{s}+(\eta^{2}-1)\alpha_{k}o(\alpha_{k}||d_{k}^{s}|{|}^{2})$. Taking expectation conditioned on *S*_*k*_ on both sides of Eq (6), we have

$${𝔼}_{k}[f(x_{k+1}^{s})]={𝔼}_{k}[f(z_{k}^{s})+\Psi_{k}^{s}(\eta )].$$ (7)

Let us denote


$$\{a_{k}={𝔼}_{k}[\alpha_{k}\nabla f(x_{k}^{s}{)}^{T}d_{k}^{s}]\leq 0,\;b_{k}={𝔼}_{k}[\alpha_{k}^{2}(d_{k}^{s}{)}^{T}\nabla^{2}f(x_{k}^{s})d_{k}^{s}],\;\epsilon_{k}={𝔼}_{k}[o(\alpha_{k}||d_{k}^{s}|{|}^{2})],$$

where $a_{k}\leq 0$ (see Lemma 4 in the following Sect 3) and for convex function $b_{k}\geq 0$. Therefore,


$${𝔼}_{k}[\Psi_{k}^{s}(\eta )]=\frac{1}{2}(\eta^{2}-1)b_{k}+(\eta -1)a_{k}+(\eta^{2}-1)\alpha_{k}\epsilon_{k}.$$

Obviously, ${𝔼}_{k}[\Psi_{k}^{s}(\eta )]$ is a quadratic function with respect to η. According to a simple calculation it can be seen that when


$$\eta_{m}=-\frac{a_{k}}{b_{k}+2\alpha_{k}\epsilon_{k}},\;and\;{𝔼}_{k}[\Psi_{k}^{s}(\eta_{m})]=-\frac{(a_{k}+b_{k}+2\alpha_{k}\epsilon_{k}{)}^{2}}{2(b_{k}+2\alpha_{k}\epsilon_{k})}\leq 0,$$

In practice, $\eta_{k}$ remains well-defined as the line search ensures $\alpha_{k}>0$ and $b_{k}\neq 0$ for valid descent directions. ${𝔼}_{k}[\Psi_{k}^{s}(\eta )]$ reaches the minimum. Considering $\eta =\eta_{m}$ in Eq (7) and since $b_{k}\geq 0$, we see that ∀k≥0

$${𝔼}_{k}[f(x_{k+1}^{s})]={𝔼}_{k}[f(z_{k}^{s})]-\frac{(a_{k}+b_{k}+2\alpha_{k}\epsilon_{k}{)}^{2}}{2(b_{k}+2\alpha_{k}\epsilon_{k})}\leq {𝔼}_{k}[f(z_{k}^{s})].$$ (8)

This leads to a potential improvement in the expected values of function *f* when the condition $a_{k}+b_{k}+2\alpha_{k}\epsilon_{k}\neq 0$ is satisfied. By combining inequalities (3) and (8), we obtain

$${𝔼}_{k}[f(x_{k+1}^{s})]\leq f(x_{k}^{s})+\rho \alpha_{k}{𝔼}_{k}[(v_{k}^{s}{)}^{T}d_{k}^{s}]-\frac{(a_{k}+b_{k}+2\alpha_{k}\epsilon_{k}{)}^{2}}{2(b_{k}+2\alpha_{k}\epsilon_{k})}\;=f(x_{k}^{s})-[\frac{(a_{k}+b_{k}+2\alpha_{k}\epsilon_{k}{)}^{2}}{2(b_{k}+2\alpha_{k}\epsilon_{k})}-\rho \alpha_{k}{𝔼}_{k}[(v_{k}^{s}{)}^{T}d_{k}^{s}]]\;\leq f(x_{k}^{s}).$$ (9)

Since ${𝔼}_{k}[(v_{k}^{s}{)}^{T}d_{k}^{s}]\leq 0$, ($d_{k}^{s}$ is a descent direction).

**Algorithm 1 Accelerated stochastic conjugate gradient: ASCG.**

**Input:** The initial point ${\tilde{x}}^{0}\in dom\;f$, the Wolfe condition parameters 0<ρ<σ<1, and epoch length *m*.

1: **for s=1,2,...,S do**

2:   $x_{0}^{s}={\tilde{x}}^{s-1}$

3:   $\nabla f({\tilde{x}}^{s-1})=\frac{1}{n}\sum_{i=1}^{n}\nabla f_{i}({\tilde{x}}^{s-1})$

4:   $v_{0}^{s}=\nabla f({\tilde{x}}^{s-1})$

5:   $d_{0}^{s}=-v_{0}^{s}$

6:   Draw samples *S*_0_ uniformly at random from {1,2,...,n}

7:   **for k=0,1,...,m−1 do**

8:     Use the Wolfe line search rule (1) to obtain the

stepsiez $\alpha_{k}$

9:     Draw samples *B*_*k*_ uniformly at random from {1,2,...,n}

10:     Compute: $z_{k}^{s}=x_{k}^{s}+\alpha_{k}d_{k}^{s}$, $g_{z}=\nabla f_{B_{k}}(z_{k}^{s})$

11:     Compute: $a_{k}=\alpha_{k}(v_{k}^{s}{)}^{T}d_{k}^{s}$, $b_{k}=-\alpha_{k}(v_{k}^{s}-g_{z}{)}^{T}d_{k}^{s}$ and

$\eta_{k}=-\frac{a_{k}}{b_{k}}$

12:     Compute:


$$x_{k+1}^{s}=\{x_{k}^{s}+\eta_{k}\alpha_{k}d_{k}^{s},\;if\;b_{k}\neq 0\;x_{k}^{s}+\alpha_{k}d_{k}^{s}\;,\;otherwise$$

13:     Draw samples *S*_*k* + 1_ uniformly at random from {1,2,...,n}

14:     Compute: $v_{k+1}^{s}=\nabla f_{S_{k+1}}(x_{k+1}^{s})-\nabla f_{S_{k+1}}({\tilde{x}}^{s-1})+\nabla f({\tilde{x}}^{s-1})$

15:     Compute conjugate parameter: $\beta_{k+1}=\frac{||v_{k+1}^{s}|{|}^{2}}{||v_{k}^{s}|{|}^{2}}$

16:     Compute: $d_{k+1}^{s}=-v_{k+1}^{s}+\beta_{k+1}d_{k}^{s}$

17:    **end for**

18:   **Option I**: ${\tilde{x}}^{s}=x_{m}^{s}$

19:   **Option II**: ${\tilde{x}}^{s}$ chosen uniformly at random from $\{x_{k}^{s}{\}}_{k=0}^{m}$

20: **end for** 21: $\hat{x}$ chosen uniformly from $\{\{x_{k}^{s}{\}}_{k=1}^{m}{\}}_{s=1}^{S}$.

**Output:** $\hat{x}$.

## 4 Convergence analysis

In this section, we mainly study the convergence rate of the proposed Algorithm 1. We start from making the following assumption for the objective function in problem (1).

**Assumption 1.** *For the objective function in problem (1):*

*(1) (**smoothness**) Function f*_*i*_ *for all* i∈{1,2,...,n} *is continuously differentiable and L*_*i*_-*smooth.*

*(2) (**convex**) Function f*_*i*_ *for all* i∈{1,2,...,n} *is convex.*

*(3) (strong convexity) The whole finite-sum function f is* μ*-strongly convex.*

These two assumptions are very common in both stochastic and deterministic convex optimizations. The Assumption 1 indicates that the whole function *f* is also *L*–smooth, where $L=\frac{1}{n}\sum_{i}^{n}L_{i}$. Below we provide a certain upper bound for the variance of gradient estimate, which are frequently used in stochastic optimization algorithms for convex optimization problems.

**Assumption 2.** *ASCG Algorithm is implemented with a step size that satisfies* $\alpha_{k}\in (0,\bar{\alpha })$ *and condition (2) with* σ∈(0,1/5).

**Assumption 3.** *There exists* $\hat{\beta }<1$ *such that*


$$\beta_{k}=\frac{||v_{k}^{s}|{|}^{2}}{||v_{k-1}^{s}|{|}^{2}}\leq \hat{\beta }.$$

*where* $v_{k}^{s}$ *is the variance reduced gradient estimator at the point* $x_{k}^{s}$ *of Algorithm 1, i.e.,* $v_{k}^{s}=\nabla f_{S_{k}}(x_{k}^{s})-\nabla f_{S_{k}}({\tilde{x}}^{s-1})+\nabla f({\tilde{x}}^{s-1})$.

**Assumption 4.** *Let* $\left\{{\tilde{x}}^{s}\right\}$ *be a sequence generated by Algorithm 1. There exists some* c∈(0,(1−σ)/σ) *such that*


$${𝔼}_{k}||v_{k}^{s}|{|}^{2}\leq c||\nabla f(x_{k}^{s})|{|}^{2}.$$

**Remark 1.** *These assumptions are standard in stochastic conjugate gradient literature [24,26,27]: Unbounded step sizes often lead to divergence in stochastic settings. Assumption 2 restricting the step size to a bounded interval is a standard and necessary practice to balance convergence speed and numerical stability. The condition* $\beta_{k}\leq \hat{\beta }<1$ *of Assumption 3 enforces that the norm of the variance reduced gradient does not grow across iterations-it reflects a reasonable decay (or controlled growth) of estimation "noise" in the gradient. Assumption 4 bounds the "quality" of the variance reduced gradient estimator $v_{k}^{s}$ relative to the true gradient* $\nabla f(x_{k}^{s})$*. Under Lipschitz continuity of f’s gradient, one can show (via variance bounds of empirical gradients) that such estimators satisfy* $𝔼\|v_{k}^{s}$ − $\nabla f(x_{k}^{s}){\|}^{2}$ *being bounded, and by Cauchy-Schwarz gradient boundedness, this leads to* $𝔼\|v_{k}^{s}{\|}^{2}$ *being comparable to* $\|\nabla f(x_{k}^{s}){\|}^{2}$*. The step size parameter* σ *in Assumption 2 is chosen such that* (1 − σ)/σ>0*, which is natural as* σ∈(0,1/5)*. Combined with the gradient related bounds from variance reduction, the constant c can be verified to lie in* (0,(1 − σ)/σ).

**Lemma 3.** *[24] Suppose that ASCG algorithm is implemented with the stepsize* $\alpha_{k}$ *that satisfies the strong Wolfe condition (2) with* 0<σ<1/2*. Then, the FR conjugate gradient method generates descent direction d*_*k*_ *that satisfy*


$$\frac{1}{\sigma -1}\leq \frac{(v_{k}^{s}{)}^{T}d_{k}^{s}}{||v_{k}^{s}|{|}^{2}}\leq \frac{2\sigma -1}{1-\sigma }.$$

**Lemma 4.** *Let* $\left\{{\tilde{x}}^{s}\right\}$ *be a sequence generated by Algorithm 1.*


$${𝔼}_{k}[\nabla f(x_{k}^{s}{)}^{T}d_{k}^{s}]\leq (\frac{c\sigma }{1-\sigma }-1)||\nabla f(x_{k}^{s})|{|}^{2}\leq 0.$$

*Proof*:


$${𝔼}_{k}[\nabla f(x_{k}^{s}{)}^{T}d_{k}^{s}]=\nabla f(x_{k}^{s}{)}^{T}{𝔼}_{k}[-v_{k}^{s}+\beta_{k}d_{k-1}^{s}]\;=-||\nabla f(x_{k}^{s})|{|}^{2}+{𝔼}_{k}[\beta_{k}]\nabla f(x_{k}^{s}{)}^{T}d_{k-1}^{s}\;=-||\nabla f(x_{k}^{s})|{|}^{2}+{𝔼}_{k}[\beta_{k}]{𝔼}_{k}[(v_{k}^{s}{)}^{T}d_{k-1}^{s}]\;\leq -||\nabla f(x_{k}^{s})|{|}^{2}-\sigma {𝔼}_{k}[\beta_{k}](v_{k-1}^{s}{)}^{T}d_{k-1}^{s}\;\leq -||\nabla f(x_{k}^{s})|{|}^{2}+{𝔼}_{k}[\beta_{k}]\frac{\sigma }{1-\sigma }||v_{k-1}^{s}|{|}^{2}\;=-||\nabla f(x_{k}^{s})|{|}^{2}+\frac{\sigma }{1-\sigma }{𝔼}_{k}||v_{k}^{s}|{|}^{2}\;\leq (\frac{c\sigma }{1-\sigma }-1)||\nabla f(x_{k}^{s})|{|}^{2},$$

*where the first inequality follows from the second Wolfe condition (2). The second inequality comes from Lemma 3. The last equality by using the definition of* $\beta_{k}$*. And the last inequality follows from Assumption 4.*

**Lemma 5.** *Suppose that Assumptions 3 holds. Then, for any k in a fixed epoch s, we have*


$${𝔼}_{k}||d_{k}^{s}|{|}^{2}\leq \phi (k)||\nabla f({\tilde{x}}^{s-1})|{|}^{2},$$

*where* $\phi (k)=\frac{2}{1-\hat{\beta }}{\hat{\beta }}^{k}-\frac{1+\hat{\beta }}{1-\hat{\beta }}{\hat{\beta }}^{2k}$.

The similar result can be see [22, Theorem 2].

In the following, we give the main property of this paper.

**Theorem 1.** *Let* $\left\{{\tilde{x}}^{s}\right\}$ *be a sequence generated by Algorithm 1. Suppose that Assumptions 1-4 hold, and* $d_{k}^{s}$ *satisfies the sufficient descent condition* $(v_{k}^{s}{)}^{T}d_{k}^{s}<c_{1}||v_{k}^{s}|{|}^{2}$ *and* $||d_{k}^{s}|{|}^{2}\leq c_{2}||v_{k}^{s}|{|}^{2}$*, where* $c_{1},c_{2}>0$*. Then the following linear convergence*


$$𝔼[f({\tilde{x}}^{s})-f(x^{*})]\leq \xi 𝔼[f({\tilde{x}}^{s-1})-f(x^{*})]$$

*holds, where* $\xi =\frac{1+\frac{L^{2}{\bar{\alpha }}^{2}}{(1-\hat{\beta }{)}^{2}}}{2Mm\mu }\in (0,1)$.

*Proof*: *If follows from the Lipschitz continuously that*


$$f(z_{k}^{s})\leq f(x_{k}^{s})+\langle \nabla f(x_{k}^{s}),z_{k}^{s}-x_{k}^{s}\rangle +\frac{L}{2}||z_{k}^{s}-x_{k}^{s}|{|}^{2}\;=f(x_{k}^{s})+\alpha_{k}\langle \nabla f(x_{k}^{s}),d_{k}^{s}\rangle +\frac{L\alpha_{k}^{2}}{2}||d_{k}^{s}|{|}^{2}.$$

*Taking expectation conditioned on S*_*k*_ *on both sides of this inequality, we have*

$${𝔼}_{k}[f(z_{k}^{s})]\leq f(x_{k}^{s})+\alpha_{k}{𝔼}_{k}[\langle \nabla f(x_{k}^{s}),d_{k}^{s}\rangle ]+\frac{L\alpha_{k}^{2}}{2}{𝔼}_{k}||d_{k}^{s}|{|}^{2}.$$ (10)

*By combining Lemma 4, Lemma 5 and (10), we have*

$${𝔼}_{k}[f(z_{k}^{s})]\leq f(x_{k}^{s})+\alpha_{k}(\frac{c\sigma }{1-\sigma }-1)||\nabla f(x_{k}^{s})|{|}^{2}+\frac{L\alpha_{k}^{2}}{2}\phi (k)||\nabla f({\tilde{x}}^{s-1})|{|}^{2}.$$ (11)

*Then, using (8) and (11), we have*

$${𝔼}_{k}[f(x_{k+1}^{s})]\leq {𝔼}_{k}[f(z_{k}^{s})]-\frac{(a_{k}+b_{k}{)}^{2}}{2b_{k}}\;\leq f(x_{k}^{s})+\alpha_{k}(\frac{c\sigma }{1-\sigma }-1)||\nabla f(x_{k}^{s})|{|}^{2}+\frac{L\alpha_{k}^{2}}{2}\phi (k)||\nabla f({\tilde{x}}^{s-1})|{|}^{2}\;-\frac{(a_{k}+b_{k}{)}^{2}}{2b_{k}}.$$ (12)

*On the other hand,*

$$\frac{(a_{k}+b_{k}{)}^{2}}{2b_{k}}\geq \frac{(\alpha_{k}^{2}Lc_{2}-\alpha_{k}c_{1}{)}^{2}||\nabla f(x_{k}^{s})|{|}^{4}}{2\alpha_{k}^{2}Lc_{2}||\nabla f(x_{k}^{s})|{|}^{2}}\geq \frac{(\alpha_{k}Lc_{2}-c_{1}{)}^{2}}{2Lc_{2}}||\nabla f(x_{k}^{s})|{|}^{2}.$$ (13)

*Substituting inequality (13) into inequality (12), we have*

$${𝔼}_{k}[f(x_{k+1}^{s})]\leq f(x_{k}^{s})+\alpha_{k}(\frac{c\sigma }{1-\sigma }-1)||\nabla f(x_{k}^{s})|{|}^{2}+\frac{L\alpha_{k}^{2}}{2}\phi (k)||\nabla f({\tilde{x}}^{s-1})|{|}^{2}\;-\frac{(\alpha_{k}Lc_{2}-c_{1}{)}^{2}}{2Lc_{2}}||\nabla f(x_{k}^{s})|{|}^{2}\;=f(x_{k}^{s})-[\frac{(\alpha_{k}Lc_{2}-c_{1}{)}^{2}}{2Lc_{2}}-\alpha_{k}(\frac{c\sigma }{1-\sigma }-1)]||\nabla f(x_{k}^{s})|{|}^{2}+\frac{L\alpha_{k}^{2}}{2}\phi (k)||\nabla f({\tilde{x}}^{s-1})|{|}^{2}.$$ (14)

*By letting* $M=\frac{(\alpha_{k}Lc_{2}-c_{1}{)}^{2}}{2Lc_{2}}-\alpha_{k}(\frac{c\sigma }{1-\sigma }-1)\geq 0$ *in inequality (14), as well as Lemma 1 and 2, we further have*


$${𝔼}_{k}[f(x_{k+1}^{s})]\leq f(x_{k}^{s})-2M\mu [f(x_{k}^{s})-f(x^{*})]+L^{2}\alpha_{k}^{2}\phi (k)(f({\tilde{x}}^{s-1})-f(x^{*})).$$

*Taking the population expectation on both sides of the this inequality, we further have*

$$𝔼[f(x_{k+1}^{s})]\leq 𝔼[f(x_{k}^{s})]-2M\mu 𝔼[f(x_{k}^{s})-f(x^{*})]+L^{2}\alpha_{k}^{2}\phi (k)𝔼[f({\tilde{x}}^{s-1})-f(x^{*})]\;\leq 𝔼[f(x_{k}^{s})]-2M\mu 𝔼[f(x_{k}^{s})-f(x^{*})]+L^{2}{\bar{\alpha }}^{2}\phi (k)𝔼[f({\tilde{x}}^{s-1})-f(x^{*})]$$ (15)

*Summing over* k=0,1,...,m−1 *and using a telescoping sum, we have*

$$𝔼[f(x_{m}^{s})]\leq 𝔼[f(x_{0}^{s})]-2M\mu \sum_{k=0}^{m-1}𝔼[f(x_{k}^{s})-f(x^{*})]+L^{2}{\bar{\alpha }}^{2}𝔼[f({\tilde{x}}^{s-1})-f(x^{*})]\sum_{k=0}^{m-1}\phi (k)\;=𝔼[f({\tilde{x}}^{s-1})]-2Mm\mu 𝔼[f({\tilde{x}}^{s})-f(x^{*})]+L^{2}{\bar{\alpha }}^{2}𝔼[f({\tilde{x}}^{s-1})-f(x^{*})]\sum_{k=0}^{m-1}\phi (k).$$ (16)

*On the other hand,*

$$\sum_{k=0}^{m-1}\phi (k)=\sum_{k=0}^{m-1}[\frac{2}{1-\hat{\beta }}{\hat{\beta }}^{k}-\frac{1+\hat{\beta }}{1-\hat{\beta }}{\hat{\beta }}^{2k}]\;=\frac{2}{1-\hat{\beta }}\frac{1-{\hat{\beta }}^{m}}{1-\hat{\beta }}-\frac{1+\hat{\beta }}{1-\hat{\beta }}\frac{{\hat{\beta }}^{2m}}{1-{\hat{\beta }}^{2}}\;=\frac{(1-{\hat{\beta }}^{m}{)}^{2}}{(1-\hat{\beta }{)}^{2}}\;\leq \frac{1}{(1-\hat{\beta }{)}^{2}}.$$ (17)

*Subtracting (17) into (16), we get*

$$0\leq 𝔼[f({\tilde{x}}^{s-1})]-𝔼[f(x_{m}^{s})]-2Mm\mu 𝔼[f({\tilde{x}}^{s})-f(x^{*})]+\frac{L^{2}{\bar{\alpha }}^{2}}{(1-\hat{\beta }{)}^{2}}𝔼[f({\tilde{x}}^{s-1})-f(x^{*})]\;\leq 𝔼[f({\tilde{x}}^{s-1})-f(x^{*})]-2Mm\mu 𝔼[f({\tilde{x}}^{s})-f(x^{*})]+\frac{L^{2}{\bar{\alpha }}^{2}}{(1-\hat{\beta }{)}^{2}}𝔼[f({\tilde{x}}^{s-1})-f(x^{*})]$$ (18)

*Rearranging (18), we have*


$$𝔼[f({\tilde{x}}^{s})-f(x^{*})]\leq \frac{1+\frac{L^{2}{\bar{\alpha }}^{2}}{(1-\hat{\beta }{)}^{2}}}{2Mm\mu }𝔼[f({\tilde{x}}^{s-1})-f(x^{*})].$$

## 5 Numerical experiments

In this section, we evaluate the practical performance of the proposed ASCG algorithm through numerical experiments on widely-used machine learning benchmarks. We compare ASCG with several state-of-the-art methods using datasets sourced from LIBSVM: svmguide1, a9a, w8a, gisette, and ijcnn1. The dataset can be obtained from https://www.csie.ntu.edu.tw/cjlin/libsvmtools/datasets/. Table 1 provides a detailed description of these datasets. All experiments were conducted in MATLAB 2017b on a 64-bit PC equipped with an Intel(R) Core(TM) i7-6700HQ CPU (2.60 GHz) and 16 GB of RAM. The experimental model is described in Sect 5.1. Sect 5.2 introduces the parameter settings corresponding to all comparative algorithms used in the experiments. The comparative performance of ASCG against other state-of-the-art methods is presented in Sect 5.3, and an analysis of the properties of ASCG is provided in Sect 5.4.

**Table 1:** The detailed description of each dataset.

Datasets	Parameters setting			
	Training size (n)	Testing sizse	Feature (d)	λ
svmguide1	3089	4000	4	1/*n*
a9a	32561	16281	123	1/*n*
w8a	49749	14951	300	1/*n*
ijcnn1	49990	91701	22	1/*n*
gisette	6000	1000	5000	1/*n*

### 5.1 The experimental model

In our experiments, we consider the following *l*_2_ regularized logistic regression for binary classification:

$$\min_{x\in {\mathbb{R}}^{d}}\{\frac{1}{n}\sum_{i=1}^{n}\log(1+e^{-b_{i}a_{i}^{T}x})+\frac{\lambda }{2}||x|{|}^{2}\},$$ (19)

where $\left\{(a_{i},b_{i})\right\}$ is coming a certain collection of dataset with $a_{i}\in {\mathbb{R}}^{d}$ and $b_{i}\in \{-1,1\}$ is the *i*th training sample and the corresponding label, respectively. λ is the regularization parameter. The regularized logistic regression model is a popular model used in the field of machine learning to measure how well an algorithm is. And it is obviously that *l*_2_ regularized logistic regression conforms to the Assumption 1 with $f_{i}(x)=\log(1+e^{-b_{i}a_{i}^{T}x})+\frac{\lambda }{2}||x|{|}^{2}$, $L_{i}=\frac{1}{4}||a_{i}|{|}^{2}$ and $L_{i}=\frac{1}{4}||a_{i}|{|}^{2}$.

### 5.2 Implementation of algorithms

We compare the proposed ASCG algorithm with several state-of-the-art conjugate gradient methods-both stochastic and deterministic-for solving the finite-sum problem (19). Specifically, the compared algorithms include: conjugate gradient with variance reduction (CGVR) [22], the stochastic conjugate gradient algorithm (SCGA) [24], and two classical deterministic conjugate gradient methods, namely Fletcher-Reeves conjugate gradient method (FR-CGM) [4] and the accelerated conjugate gradient method (ACG) [29]. The parameter settings for each algorithm are detailed below:

**CGVR**: This is the first stochastic conjugate gradient method that integrates a variance reduction technique with the FR-CGM. It involves four parameters: the Wolfe line search parameters 0<ρ<σ<1, the minibatch size *b*, and the number of inner iterations *m*. In our experiments, we set ρ=0.001, σ=0.9, $b=\sqrt{n}$, and m=⌊n/b⌋.

**SCGA**: A recent stochastic conjugate gradient method that incorporates the SAGA framework into FR-CGM. The parameters are set according to the recommendations in Table 2 of [24]: minibatch size $b=\sqrt{n}$, inner iteration count m=⌊n/b⌋, and Wolfe conditions parameters ρ=0.0001 and σ=0.1.

**Table 2:** Parameters of all comparative algorithms.

Parameter	Description	Value
|b|	The mini-batch size in CGVR, SCGA and ASCG	$\lfloor \sqrt{n}\rfloor$
ρ	The parameter of line search in all algorithms	10^−4^
σ	The parameter of line search in FR-CGM and ACG	0.9
	The parameter of line search in CGVR, SCGA and ASCG	0.1
λ	The regularization parameter in the objective function	1/n
*S*	The number of epoch in CGVR, SCGA and ASCG	50

**FR-CGM**: A classical deterministic conjugate gradient method, abbreviated as CG in this paper. It requires only the two Wolfe line search parameters. We use ρ=0.001 and σ=0.9 in all experiments.

**ACG**: A well-established accelerated conjugate gradient method developed by Andrei, which enhances convergence via adaptive step-size adjustments. Like FR-CGM, it relies solely on the Wolfe parameters. We set ρ=0.001 and σ=0.9.

**ASCG**: The proposed accelerated stochastic conjugate gradient method. Although ASCG also employs acceleration mechanisms similar to CGVR and SCGA, it involves four parameters: the Wolfe line search parameters ρ and σ (with 0<ρ<σ<1), the minibatch size *b*, and the inner loop count *m*. Our experiments use ρ=0.0001, σ=0.9, $b=\sqrt{n}$, and m=⌊n/b⌋.

For clarity, all parameter configurations are summarized in Table 2. During implementation, all feature values were normalized to the range [−1,+1]. Each algorithm was initialized from the same randomly generated starting point drawn from a standard normal distribution. To ensure fair comparison, the regularization parameter was set to λ=1/n for all methods, a common choice in machine learning applications.

In theory, we using the first Wolfe condition, i.e.,


$$f_{S_{k}}(x_{k+1}^{s})\leq f_{S_{k}}(x_{k})+\rho \alpha_{k}(v_{k}^{s}{)}^{T}d_{k}^{s}.$$

Where $S_{k}\subset \{1,2,...,n\}$. But in experiments for the compared stochastic methods, we use the standard stochastic version Wolfe condition


$$f_{S_{k}}(x_{k+1}^{s})\leq f_{S_{k}}(x_{k})+\rho \alpha_{k}\nabla f_{S_{k}}(x_{k}^{s}{)}^{T}d_{k}^{s}.$$

We refer the readers to [22,24] for the similar setting. And one can see in the later experimental results, this operation can also bring good practical results.

### 5.3 Comparison with other related algorithms

**Fig 1:**
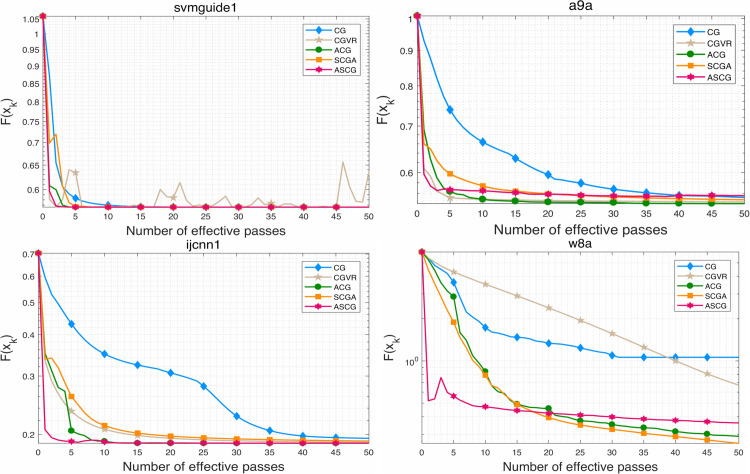
The change curve of objective function value against the number of effective passes on four datasets of different methods.

**Fig 2:**
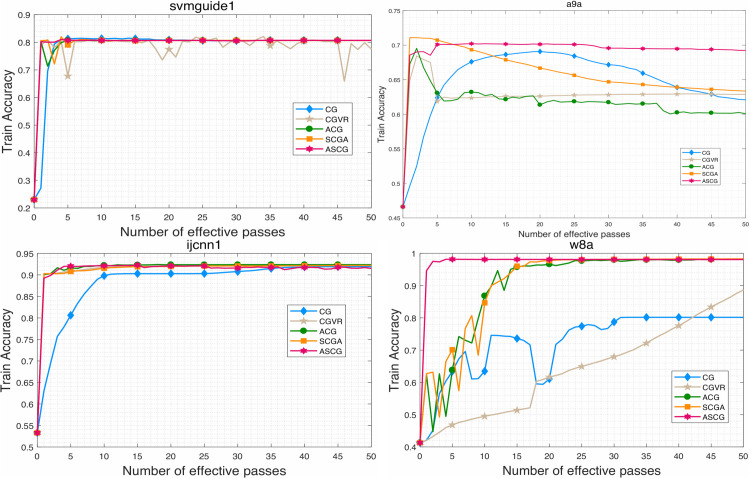
The change curve of objective function value against the gradient calculation on four datasets of different methods.

**Fig 3:**
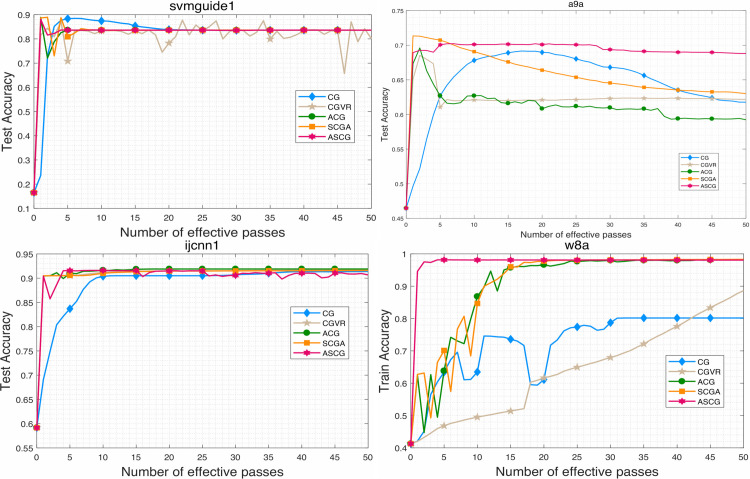
The change curve of objective function value against the gradient calculation on four datasets of different methods.

**Fig 4:**
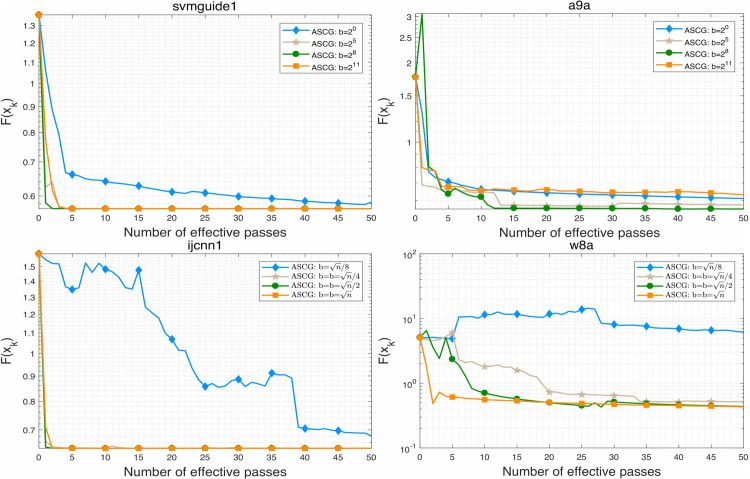
The effects of different minibatch size on objective function value of ASCG algorithm.

**Fig 5:**
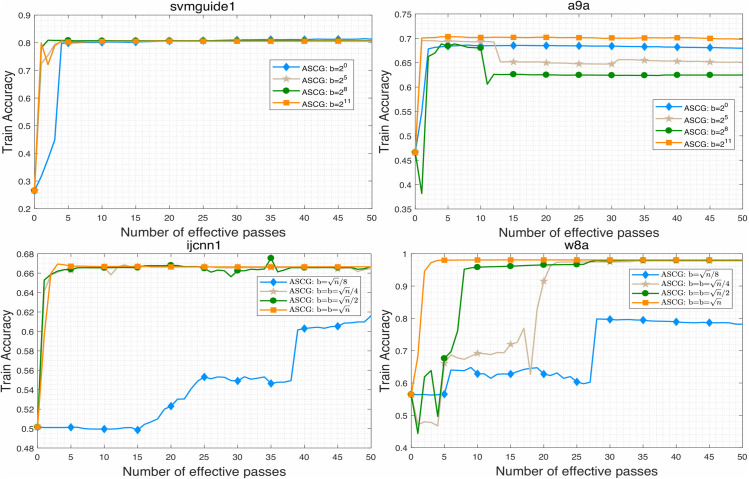
The effects of different minibatch size on training accuracy of ASCG algorithm.

**Fig 6:**
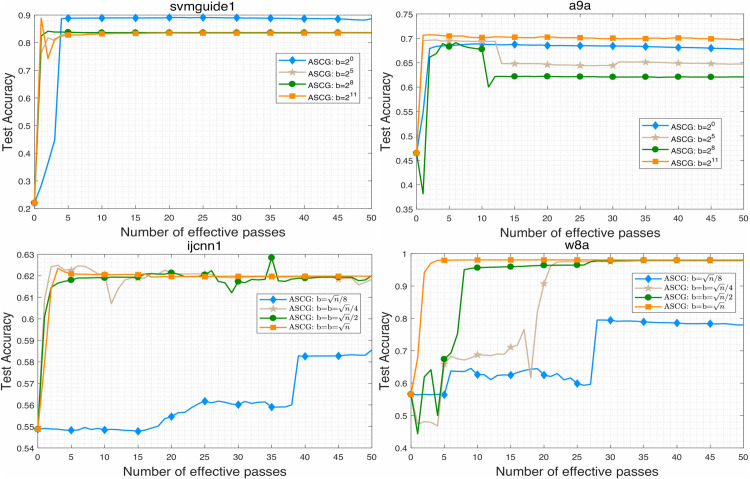
The effects of different minibatch size on testing accuracy of ASCG algorithm.

**Fig 7:**
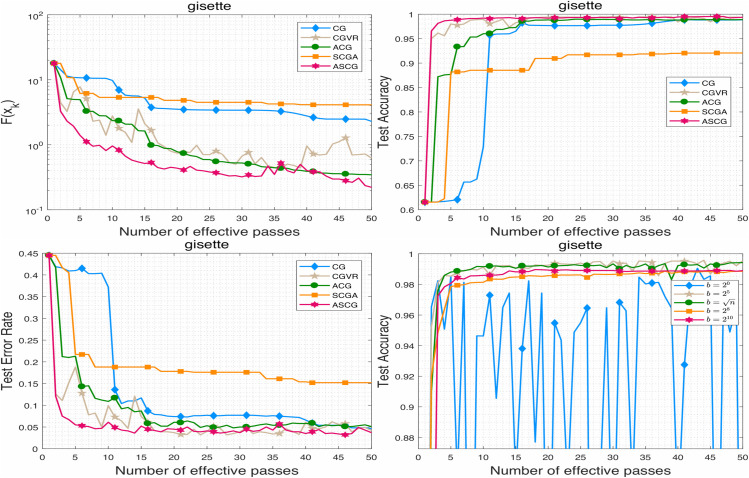
The performance of ASCG algorithm on high-dimensional dataset.

To evaluate the performance of the proposed algorithm, we compare ASCG with several competitive conjugate gradient methods for solving problem (19). The numerical results are presented in Figs 1–7. Following common practice for stochastic methods in machine learning, we measure the objective function value in terms of the number of effective passes. In these figures, the x-axis represents the number of effective passes (epochs), while the y-axis corresponds to the function value, training accuracy, and testing accuracy, respectively. Overall, the results demonstrate that ASCG performs comparably or superiorly to the other conjugate gradient methods. Specifically, variance-reduced (VR-based) algorithms consistently outperform non-VR methods. Moreover, ASCG exceeds the performance of both CGVR and SCGA, while ACG outperforms CG, empirically confirming the advantage of accelerated methods over non-accelerated ones.

In Table 3, we report the total running time, maximum training accuracy, and maximum testing accuracy of three stochastic conjugate gradient methods-each using a minibatch size of $\sqrt{n}$-across four datasets. The results indicate that the total running time of ASCG is nearly identical to that of CGVR on all datasets, whereas SCGA requires slightly more time. In general, ASCG achieves higher training and testing accuracy than the other two methods in most cases. Although CGVR and SCGA occasionally perform better, Figs 1–3 reveal that ASCG converges earlier during the initial stages.

**Table 3:** The performance of different stochastic CGM for logistic regression on four datasets.

minibatch size	Method	Total time	Maxim training accuracy	Maxim texting accuracy
svmguide1	CGVR	3.1285	82.10	87.72
	SCGA	6.2269	81.97	88.95
	**ASCG**	3.4815	81.00	88.08
a9a	CGVR	78.068	66.69	66.11
	SCGA	135.9133	69.95	69.73
	**ASCG**	53.6003	66.95	66.32
ijcnn1	CGVR	26.7859	92.28	91.68
	SCGA	109.5477	92.19	91.58
	**ASCG**	30.4645	92.28	91.60
w8a	CGVR	225.2395	92.26	92.02
	SCGA	402.9406	98.35	98.30
	**ASCG**	341.9961	98.30	98.30

To assess performance on high-dimensional data, we specifically evaluated the gisette dataset from LIBSVM. The results, shown in Fig 7, indicate that ASCG maintains superior performance even in this setting. Notably, ASCG converges faster in both objective function value and test accuracy compared to all baseline methods (CG, CGVR, ACG, and SCGA), supporting its scalability and effectiveness for large-scale problems.

### 5.4 Effect of the minibatch sizse in ASCG algorithm

In this subsection, we examine the effect of minibatch size on ASCG across all datasets. For each minibatch size, the initial step size was tuned optimally to ensure convergence. For svmguide1 and a9a, minibatch sizes of $\left\{2^{0},2^{5},2^{8},2^{11}\right\}$ were used, while for ijcnn1 and w8a, sizes of $\left\{\sqrt{n}/8,\sqrt{n}/4,\sqrt{n}/2,\sqrt{n}\right\}$ were employed. The results, illustrated in Figs 4–7, show that ASCG generally achieves better performance with larger minibatch sizes. Excessively small minibatches can lead to divergence. As shown particularly in Fig 7, the algorithm exhibits more stable and improved generalization when the minibatch size is set near $\sqrt{n}$.

## 6 Conclusion

In this paper, we propose an accelerated stochastic conjugate gradient algorithm, termed ASCG, which incorporates a variance reduction technique for solving a class of convex empirical risk minimization problems. In contrast to existing stochastic conjugate gradient methods such as CGVR and SCGA, the proposed algorithm introduces an adaptive acceleration mechanism by scaling the step size via a deflation factor, thereby simplifying the parameter selection process. We provide a rigorous theoretical analysis demonstrating that ASCG achieves an expected linear convergence rate under the Fletcher-Reeves update for strongly convex problems, along with a significantly improved expected reduction in function values. Extensive numerical experiments on four large-scale datasets, performed in the context of *l*_2_-regularized logistic regression for binary classification, confirm that the proposed algorithm outperforms several state-of-the-art methods.
